# Educating future leaders to engage the challenges of a changing world: A blended-learning approach to character and leadership education at the University of Hong Kong

**DOI:** 10.1007/s11233-024-09138-1

**Published:** 2024-09-19

**Authors:** Edward Brooks, Samson Tse, Jessie Yue Wright, Emily Burdett

**Affiliations:** 1 University of Oxford, Oxford, UK; 2 University of Hong Kong, Hong Kong Special Administrative Region, China; 3 University of Nottingham, Nottingham, UK

**Keywords:** Responsible leadership, Student leadership development, Character education, Higher education, Graduate skills, University of Hong Kong, Oxford Character Project

## Abstract

How can a new generation of students be equipped to take up positions of responsibility in a dynamic global environment, serving as leaders and citizens who will further the good of societies around the world? As the institutions responsible for educating the next generation of citizens and leaders at a formative time in their intellectual and personal development, universities have an important role to play in shaping those who will shape society. While many universities emphasize their desire to raise up future leaders for our challenging times, programmes that actively seek to help students develop qualities of character required for responsible leadership are rare. This article discusses the importance of character in leadership education and draws on a case study programme at the University of Hong Kong, which made use of a creative combination of in-person and online learning to help students grow in their intellectual understanding of leadership as well as in the self-knowledge and virtues of character required to enact responsible leadership in their own lives. The article describes the pedagogical methodology that was employed and presents the results of an exploratory, quasi-experimental longitudinal study of the programme’s impact, which was designed to establish proof of concept. The analysis of qualitative data supports the potential of such programmes to make a positive contribution to students’ intellectual understanding of leadership as well as their leadership identity and ethical formation^1^.

## Introduction

Challenges of an unstable global environment and the task of universities to educate citizens and leaders who will contribute to addressing them have combined with a focus on graduate employability to make student leadership development an increasingly prominent theme in higher education discourse (Astin, 2000; Brooks et al., 2019; Caza & Rosch, 2014; Dugan & Komives, 2007; Komives et al., 2011)**.** Universities have turned to the idea of leadership development to frame their educational mission and many have introduced student programmes, adopting a wide range of approaches (Skalicky et al., 2020). This global development in higher education has been specifically observed in discussion of university education in Hong Kong (Shek et al., 2019), which is the focus of our research. The Chinese University of Hong Kong (CUHK) aspires to: “help students to realise their potential to become future leaders in a globally connected world” (CUHK, 2022), the Hong Kong Polytechnic University (PolyU), which has a focus on servant leadership (Tong, 2019), is “committed to nurturing tomorrow’s leaders today” (PolyU, 2022), the Education University of Hong Kong (EdUHK) has a department focused on Education Policy and Leadership, and the University of Hong Kong (HKU) states in its vision: “We are committed to training and nurturing future leaders and talents equipped to tackle the grand challenges of a rapidly changing world” (HKU, 2016).

If the ambition of these universities when it comes to leadership education is clear, the practice is less straightforward. University degree programmes usually focus on specific academic disciplines which require mastery of large volumes of knowledge through specialist teaching from subject matter experts. Leadership is often taught in business schools where education is professionally focused, but it is less clear where it fits in relation to many other academic disciplines, or who should teach it. The picture is even more complicated if leadership is understood not simply as a set of skills or competencies but as involving an important personal focus (Bennis, 2009; Crossan et al., 2024). This at once brings values and character into view in leadership education and moves leadership further out of the field of vision of many traditional academic disciplines. As a result, despite the desire of universities to raise up future leaders, programmes that actively seek to help students develop as leaders and citizens who will further the good of society are often only available to a minority.

This article explores the kind of leadership education needed in the twenty-first century, focusing particularly on the importance of character. To illustrate how such education might be provided in a university context, it focuses on a case study programme at the University of Hong Kong (HKU) entitled the Character of Leadership. Launched in 2017, this programme combined in-person and online learning to help students grow in their intellectual understanding of leadership as well as in the self-knowledge and virtues of character required to enact responsible leadership in their own lives. This article also describes the pedagogical methodology that was employed and presents the results of an exploratory, quasi-experimental longitudinal study of the programme’s impact designed to establish proof of concept. The analysis of qualitative data supports the potential of such programmes to make a positive contribution to students’ intellectual understanding of leadership as well as their leadership identity and ethical formation.

### Challenges of leadership and the importance of character

In its vision statement, the University of Hong Kong directly links its aspiration to educate a new generation of leaders to the “challenges of a rapidly changing world” (HKU, 2016). Such challenges have been particularly evident in the last two years, often reaching crisis point as in the COVID-19 pandemic, global warming, civil unrest in the American Capitol, the resurgence of the Taliban in Afghanistan, and the Russian invasion of Ukraine. Hong Kong has faced its own challenges. In 2019 a proposed government bill that would have allowed the extradition of criminal suspects to China led to unrest and a violent stand-off over Hong Kong’s political autonomy and civil liberties. Mass protests and accusations of foreign interference and police brutality led to a situation described by the Central People’s Government as the worst crisis in Hong Kong since its handover to China in 1997 (Master & Pomfret, 2019). There was widespread disruption to ordinary life; the airport was blockaded; and the economy went into recession for the first time in a decade (Romei, 2019). The social unrest also precipitated widespread psychological distress with rates of depression and post-traumatic stress disorder increasing dramatically and medical experts warning of a potential mental health epidemic (Ni et al., 2020). Hong Kong’s students were heavily involved from the outset, with tertiary and secondary students numbering 3,091 of the 7,549 arrests for protest-related offences between June 2019 and March 2020 (Lau, 2020). Universities were focal points, as campuses became sites of violent confrontation between police and protesters (Kuo, 2019). The need for universities to prepare leaders for all levels and sectors of society, who are equipped with the qualities and capabilities to navigate an incredibly volatile local and global environment could not be more evident.

If this wider context points to the importance of leadership development in universities, it also speaks to the kind of leadership education that is needed. Heifetz and Linsky (2017) distinguish helpfully between technical and adaptive leadership challenges. The former are routine and require leaders to manage and optimise processes. The latter are systemic and relate to more fundamental disagreement rooted in conflicting interests, values and beliefs. The leadership challenges of late modernity are largely adaptive, with multiple strands of causation and competing values and interests that often make them highly charged and render exclusively technical solutions implausible (Grint, 2008; Heifetz & Linsky, 2017). Leaders need to be able to face such difficulties, engaging and mediating between conflicting views and values in order to find a constructive way forward even where there is no single solution. This requires a clear sense of purpose, resilience in the face of ambiguity and opposition, and a commitment to empower others and work together with diverse partners in a spirit of collaboration. These changing demands have placed an increasing focus on the values and character of those who lead (Carney, 2021; Crossan et al., 2015; Newstead & Riggio, 2023). As Mark Carney argues, leaders need to do certain things—recruit people, set priorities, initiate action—“but what ultimately most determines a leader’s effectiveness is who they are” (Carney, 2021, p. 368). Ethics and efficacy belong together, the values and character strengths of leaders are what enable them to effectively fulfil their role.

Turning to leadership development, the kind of leadership required in the twenty-first century highlights the importance of leadership education that goes beyond a focus on skills or leadership competencies (Brooks, 2021, Lamb et al., 2021a). Qualities of character (understood as reliable dispositions of thought, feeling, and action) such as courage, humility, love, and hope are at the core of good leadership (Crossan et al., 2015; Newstead et al., 2019). Two reasons make this emphasis particularly pertinent: First, many of the major challenges faced by societies around the world have an essential ethical dimension. Some shine a spotlight on the character of leaders in their origin or ongoing effect (e.g., climate change, religious intolerance, corruption, human trafficking, poverty) others cannot be addressed without selfless action (e.g., the Covid-19 pandemic) (Brooks, 2021). What is more, the well-observed breakdown of trust in leaders across sectors and societies has a fundamental character component. According to Edelman, the public relations firm known for its global trust index, “Trust is built on competence and ethics” (Edelman, 2020, p. 8). However, Edelman’s, 2020 global survey found that no sector (public, private, non-profit, media) is seen as both competent and ethical. It is not sufficient to educate a new generation of leaders, we need a new generation of responsible (competent, ethical and wise) leaders (Brooks et al., 2019; Newstead et al., 2019). Second, this emphasis accords with recent work in leadership studies on the importance of virtues of character for effective leadership (Cameron, 2011; Sturm et al., 2017) and thus as an important focus in leader development (Byrne et al., 2018; Crossan, et al., 2013; Newstead et al., 2019). This trend reconnects with a prominent historical understanding of leadership in East and West that can be traced to Confucius and Aristotle (Wang & Hackett, 2016).

The importance of a turn to character in leadership and leadership development has been championed at the university level in Hong Kong since 2010 through the service learning and servant leadership research and educational programmes of the Polytechnic University of Hong Kong (Shek et al., 2019). More recently, other Hong Kong universities have taken steps in this direction. In 2017, HKU launched the Character of Leadership programme which has developed into the university’s Lead for Life initiative. In 2020, EdUHK began to offer a Life and Character Leadership Programme for Pre-service and In-service Teachers.

### The Character of Leadership Programme

Beginning in 2017, the Character of Leadership programme was designed and delivered as an intensive two-day pilot programme for HKU students. The programme was housed in the Faculty of Social Sciences but included undergraduate and postgraduate students from across the university. It was supported by senior university faculty and industry leaders from a range of sectors.

Building on a pedagogical methodology tested over several years in the University of Oxford’s Global Leadership Initiative (Brant et al., 2020; Lamb et al., 2021a), the programme was designed to help students develop as responsible leaders who will use the opportunities, abilities and resources that they possess to further the good of their communities and wider society. Its aim was to help students grow (in modest measure in line with the length of the programme) in three important aspects of character and leadership development: (i) intellectual understanding: knowledge of what characterizes responsible leadership in different contexts, (ii) practices of leadership: habits of life that support responsible leadership, (iii) ethical formation: leadership identity (Komives et al., 2005) and virtues of character.

In 2019, a Teaching Development Grant from HKU enabled the delivery of the programme with the addition of a 3-month e-learning extension. This was intended to increase the impact of the programme by extending it over a longer period, while also testing the potential of e-learning as a cost-effective way to increase access. The programme was advertised by HKU’s Faculty of Social Sciences and through student connections and campus groups. It was open to students from across the university. Students were selected via an application process that was primarily designed to assess their engagement with the aims of the programme. Questions on the nature of good leadership and significant influences in their own thinking on the subject were designed to introduce students to central themes of the programme as well as to stimulate the kind of reflection on personal experience that can itself contribute to character and leadership development (Lamb et al, 2021a)**.** 83 students applied and 62 were accepted. All the applicants released the answers in their applications for our research, providing qualitative data to act as a baseline for a longitudinal study.

The blended programme combined a two-day intensive student forum and a three-month e-learning extension programme. The e-learning component was mediated through HKU’s Moodle platform in conjunction with the use of the WhatsApp personal messaging service to engage participants and post reminders. The overarching aim was to help students in the development of character virtues necessary to function as ethical and effective leaders, able to take initiative and get things done in their own context in a way that achieves operational goals, enables others around them to flourish, and furthers the good of their community or organisation. The theoretical framework for the programme was drawn from a neo-Aristotelian approach to character development (Kristjánsson, 2015) that has been utilised elsewhere in leadership development (Brooks et al., 2019; Contreras, 2023) and has parallels with the Confucian tradition of character formation (Wang & Hackett, 2016). This framework for character development has a number of associated pedagogical strategies, which have been applied to student character and leadership development programmes in other contexts: “i) habituation through practice, ii) reflection on personal experience, iii) engagement with virtuous exemplars, iv) dialogue that increases virtue literacy, v) conversations about situational variables, vi) moral reminders that make norms salient, vii) friendships of mutual accountability” (Lamb et al., 2021a). These methods were integrated and deployed in multiple ways through the programme, which adopted a “flipped classroom” approach, relying heavily on facilitated discussion of pre-reading material, engagement with experienced practitioners, peer-learning and guided self-reflection.

The programme was structured around five themes: the nature of good leadership, the concept and importance of character, practices of leadership, the power of situational pressures and institutional dynamics, and the relationship between leading and following. It had a particular focus on six specific character virtues: service, gratitude, honesty, humility, wisdom, and pro-social purpose. These were chosen for five reasons: (i) They were identified in a review of leadership studies literature as important for good and wise leadership (Chun, 2005; Crossan et al., 2015; Gini, 2013; Haskins et al., 2018; Kempster et al., 2011; Sturm et al., 2017; Wright & Quick, 2011); (ii) they are especially relevant for leadership development during the life stage of emerging adulthood (Arnett, 2014, 2018; Brooks et al., 2019); (iii) they are recognised as important by a wide range of moral traditions**;** (iv) many have an important de-centring effect, shifting the focus away from narrow self-interest and towards public service and the character required for positive influence and impact in a pluralistic global context (Lamb et al., 2021a); (v) they provide a substantive focus to the programme without being unduly restrictive, giving sufficient structure while also allowing each student to develop along their own personal path of leadership formation.

The student forum took place over two days and included: pre-readings on different aspects of leadership from a variety of sources, including philosophy, poetry, personal correspondence, and contemporary opinion articles; facilitated discussions with groups of around eight students, chaired by university faculty and experienced leaders from consulting, entrepreneurship, finance, law, media, and the military; interactive talks on the nature of good leadership, the nature and importance of character, institutional pressures and incentives, and developmental practices; peer-learning exercises; student presentations; talks from guest speakers including the Pro-Vice Chancellor for Teaching and Learning at HKU and a former Executive Director of a global investment bank; a panel discussion with senior corporate executives discussing the role of character in leadership in their personal experience, commercial institutions and social impact work; and a series of creative workshops, drawing on rhetoric, drama and jazz to engage themes such as failure, friendship and advocacy. Across the two days there was time set aside for personal reflection and informal opportunities for conversation with peers, speakers and facilitators during breaks and over shared meals.

The e-learning programme began shortly after the forum. It was delivered via HKU's Moodle platform and comprised ten modules (featuring 9 videos, 8 external audio or video resources, 18 carefully selected readings, and 10 personal reflection exercises) spread evenly over three months. The first two modules returned to conceptual and practical questions on the nature of leadership and importance of character. Subsequent modules focused on specific virtues with modules on altruism, honesty, humility, purpose, courage, and wisdom. There were also modules on following and failure. The e-learning programme was intended to build on the student forum, exploring character and leadership in a way that engaged with students’ leadership roles in their own communities and contexts (e.g. internships, part-time jobs, student societies, community roles). An important part of the programme’s design was a “self project” (Berkowitz, 2021), whereby students were encouraged to identify one particular character virtue that is important for leadership in their own context and which they wanted to make a personal focus. Drawing on the concurrent experience of a leadership setting (formal or informal) alongside the programme that could function as a good source of reflection and a setting for the application of new ideas and practices. This context would be a practical site for character and leadership development, drawing wider experiences and learning opportunities into each student’s journey of personal development, as well as facilitating students’ ownership of their developmental goals. The intention was to help students develop as reflective practitioners and cultivate practical wisdom (Schwartz & Sharpe, 2010), with both a growing intellectual understanding of what makes for good leadership and a growing practical understanding of how character-based leadership is applied in a range of situations and circumstances.

The first seven modules followed a similar structure, comprising an introductory video and explanatory narrative; conceptual content in the form of a written text or third-party video on the theme of the module; reflection questions connected to the content; a short reflective writing task relating the theme of the module to students’ own particular context; and a “self project” (Berkowitz, 2021) component involving personal exploration or habituation of a chosen leadership virtue. Students were asked to contribute written reflections to an online forum and to contribute questions or comments in response to posts by other students. Following a review of student engagement, including several phone interviews with participants, the amount of content in the final three modules was reduced. A short paragraph by way of introduction was followed by a short video and article on the theme of the module and an invitation to join a moderated discussion on the programme’s WhatsApp group. Throughout the programme, contributions were moderated by the course leader, who included additional comments and questions. Students were also engaged via an email list and via WhatsApp, informing them when new modules were posted and encouraging them to contribute.

We hypothesized that: (i) the programme would help students to develop in three dimensions of responsible leadership (intellectual understanding, practices of leadership, ethical formation) revealed by both quantitative and qualitative data, (ii) the e-learning component would extend and deepen the impact, and (iii) students would exhibit modest growth compared to a control group of their peers at HKU who did not participate in the programme.

## Methods

**Fig. 1:**
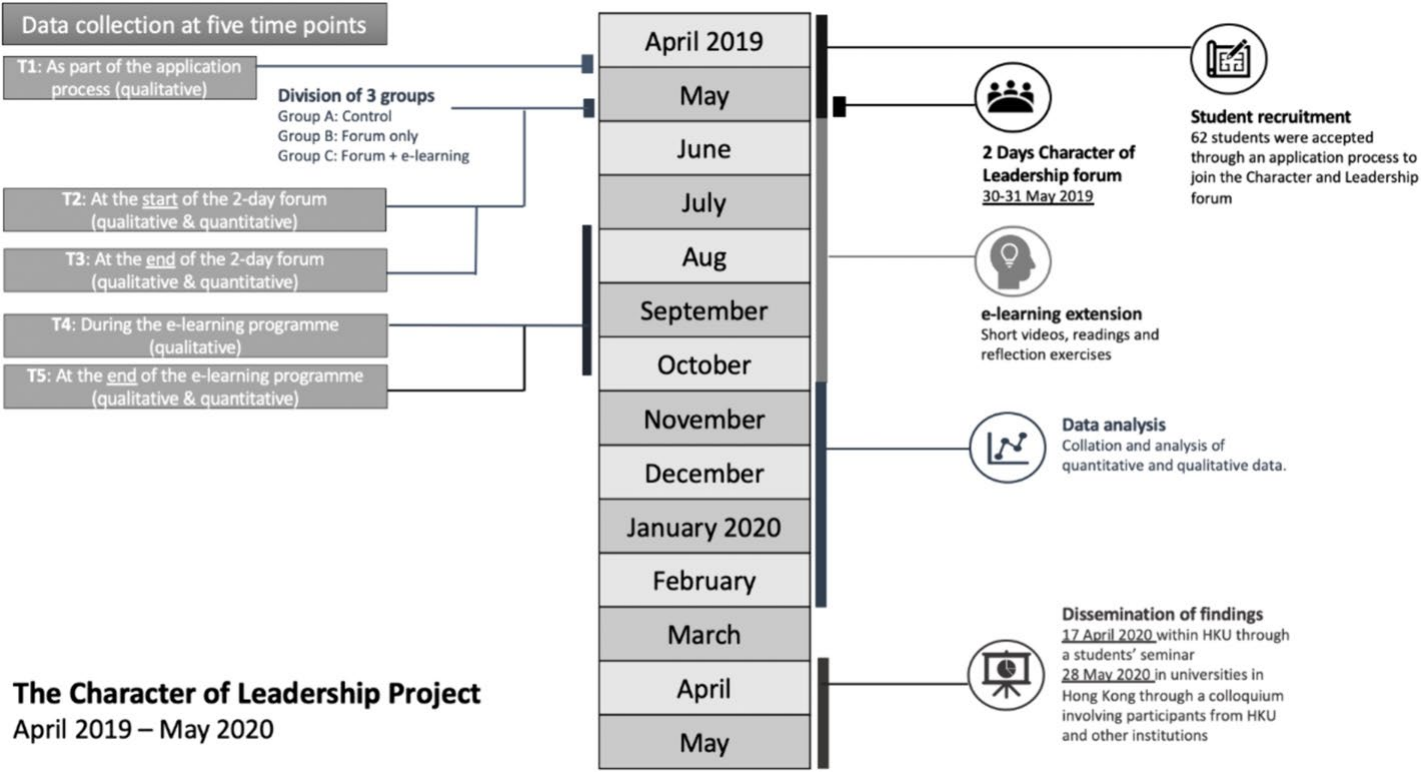
Project timeline

The study involved a mixed-method pre- and post-test quasi-experimental research design involving three groups: no intervention (group A); 2-day programme only (group B); and a 2-day programme and e-learning extension (group C). Quantitative (psychometric) and qualitative survey data were gathered at five points: T1, open-answer survey data (n = 62) was collected as part of the application process; T2, psychometric survey data (intervention n = 60; control n = 29) was collected at the start of the 2-day forum; T3, psychometric survey, open-answer survey and Likert-scale data (intervention quant. n = 40, qual. n = 48; control n = 13) was collected at the end of the forum; T4, interview data (n = 4) was collected during the e-learning programme; T5, psychometric survey, open-answer survey and Likert-scale data (intervention quant. n = 13, qual. n = 12; control n = 8) was collected at the end of the e-learning programme. Ethical approval was obtained from the Human Research Ethics Committee of HKU (Fig. 1).

Psychometric data were collected through two surveys, administered in a single online questionnaire at T2, T3 and T5. The first was a 59-item survey, combining validated psychometric self-report measures, with compatible question formats and response scales, relating to 6 of our focal virtues: Self-Report Altruism Scale by (Rushton et al., 1981); Gratitude Scale by (McCullough et al., 2002); items relating to honesty from the HEXACO-60 Scale by (Ashton & Lee, 2009); Humility Scale by (Elliott, 2010); Brief Calling Scale by (Dik et al., 2012); Abbreviated 3-Dimensional Wisdom Scale by (Thomas et al., 2017). The second survey was the 96-item VIA IS-M survey (McGrath, 2017), a shortened form of the full 240-item VIA-IS measure, which balances psychometric accuracy with test-length, measuring a broad group of 24 character virtues. The VIA-IS is widely used as a self-report measure in character education.

Alongside the psychometric surveys we made use of open-answer and Likert scale questions to gain a deeper understanding of students’ experience over the course of the study. In order to participate in the programme, students were required to complete an online application form comprised of open-answer questions regarding their understanding of leadership, personal leadership experience, what they hoped to get out of the programme, and future aspirations. Gathered in advance of students’ participation in the programme (T1), this data provided a useful baseline. Students were also asked to respond to open-answer and Likert-scale questions in questionnaires administered after the 2-day Forum (T3) and e-learning programme (T5). Four participants in the e-learning programme, selected at random, were interviewed over the phone midway through the e-learning.

The combination of qualitative and quantitative methods in our research design, and in character education research more widely, reflects the particular challenges involved in measuring character development (Duckworth & Yeager, 2015; Kristjánsson, 2015). While no single measurement approach has been developed that can reliably encompass all aspects of character development, the combination of methods allows for numerical and narrative approaches, representing divergent epistemological stances, to be productively integrated. The depth of analysis through qualitative methods can illumine and support the focused and arguably more objective results delivered by psychometric surveys. This combination is particularly helpful in small studies, where qualitative data are easier to gather and process and achieving a sample size sufficient for well-powered quantitative analysis is difficult.

### Participants

Students at HKU were informed of the programme via faculty email lists, word of mouth and campus posters, and invited to submit an application through an online form. Capacity was limited, requiring selection of 62 out of 83 applicants based on engagement exhibited in the application (those who were not selected provided only cursory answers). Of the selected students, 48 were undergraduates in their first three years of study and 14 were masters or PhD students; 37 were female and 25 were male; and they came from 11 countries, with 26 from mainland China and 21 from Hong Kong. These students all participated in the in-person forum, where they were allocated to eight facilitated groups in order to enable small group discussion, sharing and peer learning. Effort was made to ensure diversity with respect to gender, nationality, and year of study in each group. Four of these groups (30 students in total) were selected at random to participate in the e-learning extension programme. Those not selected were given access to the programme once the study was completed. They formed a control group for the e-learning programme. An additional control group of 29 students who did not participate in any part of the programme was also recruited. This latter group was composed of volunteers from the HKU student body, who responded to a faculty email asking for volunteers to support a study of “personal attributes amongst HKU students.” Volunteers did not know they were a control group for the programme.

## Results

### Quantitative results

#### i. Six character virtues

We first examined participant responses for our six focal character virtues: gratitude, honesty, humility, service, vocation and wisdom. We created an index for each virtue by grouping the items into separate indices and providing an average score for each virtue, at each timepoint, for all groups, according to each participant.

To evaluate whether responses from individuals in the treatment groups (B and C) were different compared to their baseline/pre-treatment scores and compared to the control group (A), we used six 3 × 3 repeated measure ANOVA’s to examine scores between group (3: A, B, C) by time (3: T2, T3, T5) for each virtue. We did not find any significant differences across time, between groups, or any interactions of group by time for any virtue, *p*s > 0.05. However, there were some upward trends. For example, responses for honesty and humility showed that ratings increased over time, especially for the treatment groups (B and C). It is worth noting that there may have not been enough power to detect significant results because of a high participant dropout from T2 to T5. This meant that the sample size from which we were able to collect results across all three time points was low, with only 8 participants in the control group (A), 6 participants in the forum-only group (B), and 7 participants in the forum and e-learning group (C).

Because of the low sample size at T5 and the loss of participants that could be included in a repeated measures analysis, we also compared the control group (A) with both treatment groups (B + C) at T2 and T3. At T3 both treatment groups would have received the same treatment. We ran six 2 × 2 repeated measures ANOVA’s for each virtue with group (2: A and B + C) across time (2: T2 and T3). There were no significant differences between groups, or any interactions,* p*s > 0.05. There was one significant difference across time periods for vocation, F(1,55) = 5.97, p = 0.018. Participants had significantly higher scores at T3 than T2. Sample size was still low with 13 participants in the control group and 40 in the treatment groups.

#### ii. VIA-IS-M

We then examined participant scores for each of 24 virtues within the VIA measure: appreciation of beauty, bravery, creativity, curiosity, fairness, forgiveness, gratitude, honesty, hope, humour, judgment, kindness, leadership, love of learning, love, humility, perseverance, perspective, prudence, self-regulation, social intelligence, spirituality, teamwork, and zest. We created indices for each virtue by grouping items and creating an average score for each time period and person.

To evaluate change in responses over time we conducted 24 3 × 3 repeated measures ANOVA’s for each virtue with group as the between measure (3: A, B, C) by Time (3: T2, T3, T5). We did not find any significant differences across time, between the groups or any interactions, for any of the virtues, *p*s > 0.05. Similar to the six virtues above, there may not have been sufficient power because of low sample sizes: counting only those who provided data across all three time points, there were 8 participants in the control group (A), 6 participants in the forum-only group (B), and 7 participants in the forum and e-learning group (C). If we examine trends, responses for curiosity increase at the second time period. Also, responses for fairness in group C increased between T2 and T3. Responses for humility increased for both treatment groups (B and C) over all of the time periods.

We also ran further 2 × 2 repeated measures ANOVA’S to compare change in responses from T2 to T3. Again, we combined both treatment groups (B + C). We thus examined group (2: B + C and A) across time (2: T2 and T3). Again, we found no significant differences across time, between groups or any interactions, *p*s > 0.05.

In summary, comparing programme participants to a control group, we found no statistically significant changes. However, there were indicative upward trends for programme participants compared to a control group when it came to curiosity, fairness and humility. Low sample size, and a higher than anticipated drop-off rate impacted analytical power and made the detection of significance difficult.

### Qualitative results

Qualitative data were thematically coded, using a combination of “in vivo” coding (using the vocabulary supplied by students), process coding (using “-ing” words as codes in order to capture action), and descriptive coding (using nouns that summarize content). A coding framework was established to discern student development according to the aims of the programme: (i) Intellectual understanding of good leadership: we examined how students understood good leadership and how this changed over time; (ii) Practices of leadership: we sought to identify practices or habits that support character and leadership development (e.g. reflecting on experience, identifying and emulating exemplars); (iii) Leadership identity and ethical formation: we investigated the data for insight into how students think of themselves when it comes to leadership and virtues of character relevant to good leadership that they actively embody or are seeking to cultivate.

Two researchers discussed and agreed the coding methodology, working through a sample of data together in order to establish a provisional list of codes and a common approach. Data was then coded by one researcher and reviewed by the other. Codes were thematically grouped in an iterative process and the results were evaluated and discussed by the same two researchers. A final step identified illustrative quotations and used codes to calculate relevant percentages.

Overall, the programme was very well received. Asked in an anonymous post-programme questionnaire to “sum up” their experience of the forum (T3 qual. n = 48), comments were almost entirely positive (96%). Participants described it as “inspiring” (22%) and “insightful” (24%). Others used such language as “refreshing”, “impactful”, “fruitful”, “profound”, “a real game changer”. They gave the forum sessions an average rating of 4.2 on a 5-point Likert scale. Students especially appreciated the nature of the learning environment, which was described by one student as “a community where you can safely share your ideas and grow together” [student B14]. Others highlighted the opportunity to learn from a diverse group of peers, as well as facilitators and guest speakers, who were experienced practitioners from a range of academic and professional backgrounds. The presence of this group was particularly appreciated, with the input of facilitators rated as “very good” or “excellent” by 96% of students.

Students who engaged with the e-learning course also generally appreciated it. Asked to sum up their experience (T5 qual. n = 12), 58% provided positive or strongly positive responses, 17% responded neutrally, 25% responded negatively. Positive responses paralleled the language used by students to sum up their experience of the in-person forum. Students described it as “inspirational”, “enlightening”, “an eye-opening and nourishing experience”, and “a fruitful and rewarding experience”. Neutral responses described the programme as “detailed” and “high-quality” but flagged the lack of interactivity and their inability to complete the programme. This was further highlighted in negative responses, which also identified technical challenges in accessing the online platform. Circumstantial and technical challenges, discussed below, as well as common difficulties when it comes to e-learning pedagogy, seem to have been an important factor in a low overall level of engagement with the e-learning course.

Here we present the results of qualitative analysis, distinguishing between the two parts of the programme (in person and e-learning):

#### i. Intellectual understanding of leadership

In the baseline data (T1 n = 62), students consistently emphasized leadership as a formal position of power and authority. To be a “real leader” is to be formally “in charge”. When speaking of their own experience they referred to titled roles in rigid, hierarchical structures in student societies. For example, a typical response on the nature of leadership referred to a student’s position as “External Vice Chairperson of University Hall Students’ Association, HKU Students Union” [student A14]. On this view the leader is the person who occupies a specific role as “president”, “chair” or “executive”. Their authority is accompanied by specific organizational responsibilities that they need to fulfil.

Following the Forum, 92% (44 out of 48 respondents) reported positive growth in their understanding of leadership as a result of their participation in the programme. There was a strong trend in the data towards a more dynamic, relational and service-oriented view of leadership: “My understanding has become more dynamic as leadership is a relational and situational concept” [student F45]. “I used to think that a leader is in some way a dictator, but now I come to realize that a leader doesn’t dictate… but brings out the best of the people around them” [student F37]. “Leadership is more like serving others, bringing the potential and best from others” [student F3].

#### ii. Practices of leadership

From the baseline data (T1), it is clear that many students were active in seeking leadership opportunities in student societies and voluntary positions. Some students identified personal developmental activities relating to leadership. These included: reading, attending talks, observing experienced leaders, mentoring, attending programmes.

Following the 2-day Forum (T3), 17% of students (8 out of 48) described a new commitment to practices that support good leadership, such as listening and reflection. Several students highlighted a new motivation when it came to ongoing leadership development. As one student put it, “I am very motivated now to better myself. I also have new insights and directions to practice in the future, no matter personally or as a leader role” [student F28].

Following the e-learning programme (T5), 12 students responded to a final questionnaire. 75% of these students reported new or sustained practices of leadership development. 50% of students reported regular reading, 33% watching video content, and 33% reported practices of reflection including journaling. 66% reported additional leadership learning activities (reading articles, watching TED talks, joining a mentoring programme) beyond the limits of the programme.

#### iii. Leadership identity and virtues of character

The baseline data (T1) indicates that prior to the programme, students understood leadership as a position of authority in an organization. They took themselves to be leaders in accord with the roles they possessed, many carefully listing the various positions they had held. The idea of everyday leadership “not defined by title or power” [student A36] was evident in less than 5% of responses (3 out of 62). Leadership was something students aspired to: “in the future, I will become a leader somewhere” [student A17]. While some students (32%) expressed their desire to lead in terms of personal interest or ambition, far more (68%) expressed a clear pro-social purpose. This was evident in students with a wide range of academic backgrounds and vocational ambitions. It was linked to the idea of citizenship with many students echoing the desire to “truly contribute to the society” [student A68], specifically identifying the needs of Hong Kong or their home country.

Following the Forum (T3), the change in understanding of leadership from a formal position of authority to a “relational and situational concept” where leaders “bring out the best” of those around them was accompanied by students reporting growth in confidence and “self-belief”. Students reported a sense of personal growth over the two days with 19% of students (9 out of 48) identifying a stronger others-centred orientation, expressed in the idea of “giving” or “service”.

Following the e-learning programme (T5), 75% of students (9 out of 12 respondents) reported personal development related to ethical leadership over the programme as a whole. Students reported growth in “confidence” and self-acceptance when it came to failure. They also grew in personal awareness that they could be both leader and follower. They reported growth in particular character virtues and capacities including humility, kindness, empathy, gratitude, open-mindedness, optimism, initiative, confidence, and growth mindset.

## Discussion

Major societal challenges and a wider global context of complexity and uncertainty present important challenges and opportunities for higher education. Amongst them is the need for universities to develop “the competencies and qualities their students will need to succeed and flourish in their careers and help our society meet the challenges it faces” (Bok, 2020, p. 159). While this points to the importance of character and leadership development in universities, educating for character in modern higher education is not straightforward. Derek Bok (2020) highlights the need for empirical studies to evaluate new approaches and establish best practice when it comes to the pedagogy and measurement of character and skill development in universities. To date there have been some empirical studies of character development in higher education in the USA, Europe, and Asia (Brant et al., 2020; Dufresne & Offstein, 2012; Hilyana & Hakim, 2018; Lamb et al., 2021b; Leesen & Van Lenning, 2022) and a recent consultation of higher education academics and administrators from 11 universities around the world led to the production of a proposed framework for *Character Education in Universities* (Jubilee Centre for Character and Virtues & the Oxford Character Project, 2020). However, theoretical and empirical work on character and leadership development in universities is an emerging field.

As a contribution to this area of inquiry, both the strengths and limitations of the study are important. Analysis of quantitative psychometric data indicated a significant increase in the sense of vocation for forum participants and positive trends in curiosity, fairness, honesty and humility. This is promising given the contribution of these virtues to important aspects of leadership such as ongoing learning (curiosity), acting with justice (fairness, honesty), maintaining an appropriate sense of self (humility), and motivation beyond the self (vocation). Humility, in particular, is singled out in organisational leadership literature as a seminal virtue (Morris et al., 2005; Rego et al., 2017) with experimental data indicating its contribution to team performance and employee resilience (Zhu et al., 2019). However, relatively small numbers in the study and a high drop-off rate left us with low statistical power, resulting in quantitative analysis that was indicative but inconclusive. While specific contextual factors played a part in participant drop-off, the challenges faced in quantitative analysis point to the wisdom of seeking larger sample sizes in future self-report survey studies. There may also be value in other quantitative methods, such as experience sampling (Csikszentmihalyi & Larson, 1987), which may not require large sample sizes and can be conducted through a mobile phone app (van Berkel et al., 2017).

Analysis of qualitative data provided deeper insight into the effect of the programme, underlining the value of mixed-methods research in relation to character and leadership development. The two-day forum was effective in introducing a new idea of leadership, focused less on positional authority and more on the ideas of service and bringing the best out of others. It was impactful when it came to the motivation to adopt developmental practices and some students (19%) reported personal growth in terms of increased service orientation. However, the adoption of new practices of leadership and growth in leadership identity and character require a longer period of time, which was the motivation behind the additional e-learning component. Indeed, students who were motivated to complete the e-learning programme reported encouraging levels of growth in both practices of leadership and leadership identity and character. Our analysis suggests that the Character of Leadership programme helped students to develop in their understanding of and commitment to responsible leadership, but the main limitation was that only a small number persevered through the e-learning programme.

Learning from the programme as a pilot study, the value of in-person and relational educational methods for the development of leader character was underlined. The e-learning component was less successful but provides lessons to take forward into future hybrid or online programmes. There were four particular challenges: First, the timing of the programme in the summer vacation proved less conducive to student engagement than we had anticipated. Students were away from campus and did not respond readily to contact via their university email addresses. Second, some students faced technical difficulties accessing the university’s online platform and required technical support before they could begin the programme, a hurdle that diminished engagement at the outset. Third, students engaged with online content as a “repository of resources and information” (Henderson et al., 2017, p. 1571) but neglected the interactive elements of the programme. Our efforts to maintain the personal approach of the 2-day programme through peer-learning, message boards, and exercises with written mentor feedback encountered a lack of “task-technology fit” (Al-Samarraie et al., 2018, p. 2014). Fourth, there was severe social upheaval in Hong Kong during the period of the study. Widespread social unrest, which involved many university students, began on 4th June 2019, four days after the 2-day forum and right at the point where we were seeking to maximise student engagement in the e-learning component of the programme. It is hard to judge the impact of these events on participation but it is important to bear in mind the extraordinary circumstances that prevailed through the research period.

Learning from the challenges we faced can enable the better use of e-learning methods in similar programmes in the future. Upon reflection, it has become evident that we may have over-estimated the level of student engagement without the support of facilitators and the positive group dynamics that arise from personal interaction with other learners. However, it is crucial to acknowledge the significant, far-reaching, negative impact of the social unrest that prevailed in Hong Kong during the research period. A central recommendation for future programmes would be to adopt a more active facilitation methodology for online learning. For example, a programme might make online resources available via a virtual learning environment but provide the relational backbone through group video calls and discussions, along with individual tutorials to set goals and discuss progress. Such an approach plays to the strengths of many online platforms, which are useful repositories for content but limited in the user-experience when it comes to the kind of interpersonal engagement that was highly valued in the in-person forum. 

The contrast in engagement between in-person and online elements brings practical challenges of online learning to the fore, but it also highlights the strong engagement in the first part of the programme. Student perceptions of leadership evident in qualitative data underline that the aspiration of universities to educate “citizen-leaders” (Harvard College, 2023) who will serve society is closely aligned to the education desired by students themselves. While critics may caution that character development programmes in universities represent unwarranted imposition on students (Carr, 2017) and that “citizenship formation is not our job” (Fish, 2017), the Character of Leadership programme is part of a growing body of evidence (e.g., Brooks et al., 2019; Callina et al., 2023; Lamb et al., 2021b; Shimer, 2018) pointing to the appetite of students for university programmes that not only enable them to learn leadership skills but explore and develop their character and purpose. 

## Conclusion

This article has argued that institutions of higher education should support their oft-stated desire to raise up a new generation of leaders for challenging times with an approach to leadership development that focuses on responsible leadership, educating students to lead in a way that furthers the good of society. It has presented and analysed a specific approach to character and leadership development amongst students at one university, evaluating the results of a controlled, longitudinal study in order to establish proof of concept. It has shown, through the analysis of qualitative data, that such a programme was welcomed by students and furthered their intellectual understanding of leadership, commitment to practices of good leadership, and leadership identity. Finally, it has highlighted ways in which such programmes might be further developed.
